# Noninvasive Detection of Acute Hyperglycemia Using Signal from Wearable ECG Sensors Considering Individual HRV Response Delays to Glucose

**DOI:** 10.3390/bios16050251

**Published:** 2026-04-29

**Authors:** Jiho Ha, Ho Bin Hwang, Hayoung Kim, Seungyeon Lee, Jeyeon Lee, Jung Hwan Park, Jongshill Lee, In Young Kim

**Affiliations:** 1 Department of Electronic Engineering, Hanyang University, Seoul 04763, Republic of Korea; hajiho24@hanyang.ac.kr (J.H.);; 2 Department of Biomedical Engineering, Hanyang University, Seoul 04763, Republic of Korea; hobin0215@hanyang.ac.kr; 3 Division of Intelligent Robot, Daegu Gyeongbuk Institute of Science and Technology, Daegu 42988, Republic of Korea; 4 Department of Biomedical Engineering, College of Medicine, Hanyang University, Seoul 04763, Republic of Korea; 5 Department of Internal Medicine, College of Medicine, Hanyang University, Seoul 04763, Republic of Korea

**Keywords:** blood glucose, electrocardiogram, heart rate variability, hyperglycemia, deep learning, physiology, diabetes, biomedical engineering

## Abstract

Noninvasive blood glucose monitoring is crucial for detecting early dysglycemia, yet continuous glucose monitors remain invasive and costly. Electrocardiogram (ECG) and its derived heart rate variability (HRV) measure may offer a noninvasive indicator of autonomic and cardiac responses associated with acute changes in glucose. In this study, 30 adults underwent a 75 g oral glucose tolerance test with concurrent ECG Holter and interstitial glucose monitoring. From these recordings, HRV and ECG features were extracted. A deep learning classifier with HRV and ECG was then trained to detect hyperglycemia (glucose ≥ 180 mg/dL). Cross-correlation analysis confirmed a significant association between HRV and glucose (Pearson r ~0.65, *p* < 0.05) when aligning each participant’s data according to individual response delays. The model achieved high classification performance under rigorous temporal validation (accuracy ~89%, area under the receiver operating characteristic curve ~0.89). Saliency analyses revealed that the classifier’s decisions focus on distinct ECG waveform transitions and key HRV features linked to glucose-induced autonomic changes. Overall, acute hyperglycemia elicited discernible changes in HRV and cardiac conduction, supporting the feasibility of this physiologically grounded approach for detecting the acute hyperglycemic phase under controlled conditions. This method holds promise for real-time implementation in wearable devices, enabling early diabetes risk screening.

## 1. Introduction

Blood glucose is a key physiological indicator of metabolic homeostasis, interacting closely with multiple systems, including the autonomic nervous system (ANS), the endocrine system, and the cardiovascular system. As the body’s capacity for glucose regulation declines, diabetes mellitus develops, resulting in chronic hyperglycemia [1,2]. Prolonged hyperglycemia is a principal driver of severe complications, including cardiovascular disease, kidney disease, stroke, blindness, and limb amputation [1,2]. Unfortunately, in many individuals, diabetes is only diagnosed after substantial progression of metabolic dysfunction.

Traditionally, blood glucose has been assessed by finger-stick capillary sampling. Finger-stick testing is inexpensive and accurate, but each measurement is painful and provides data for discrete time points. In contrast, continuous glucose monitoring (CGM) systems enable near-continuous glucose measurement; they have demonstrated improved diabetes management across multiple studies and been established as the standard of care [3,4,5,6]. However, current CGM devices still require invasive sensor insertion and remain costly, which limits use. For these reasons, individuals not living with diabetes are unlikely to adopt CGM for preventive purposes [7,8].

Noninvasive blood glucose monitoring has attracted sustained interest in recent decades. Multiple noninvasive modalities have been proposed, including Raman spectroscopy, fluorescence-based sensing, optical coherence tomography, and optical polarimetry. These optical approaches are sensitive to ambient conditions and require costly instrumentation, which limits their suitability for continuous monitoring in daily life. To address these constraints, prior studies have leveraged physiological bio-signals to estimate and analyze glucose dynamics [9,10,11]. Moreover, recent advances in soft and wearable biosensing platforms have highlighted the potential of noninvasive, continuous monitoring technologies for personalized health assessment in daily life [12]. In parallel, advances in soft bioelectronic electrodes have improved the feasibility of high-fidelity wearable electrophysiological monitoring, including under dynamic conditions, where motion artifacts and instability at the skin–electrode interface remain key technical challenges [13]. Among these strategies, the electrocardiogram (ECG), which monitors electrophysiological signals of cardiac origin, and derived heart rate variability (HRV) are relatively easy to record, transmit, and process, and can be measured continuously using wearable devices [14]. Given their accessibility and noninvasive nature, ECG- and HRV-based monitoring has emerged as a promising approach for diabetes management and early risk detection in vulnerable populations.

Many investigators have examined associations between blood glucose and both cardiac electrophysiology and autonomic responsiveness. Two mechanistic avenues have been reported: (i) ECG morphological changes driven by alterations in myocardial potassium ion (K^+^) conductance, and (ii) modulation of autonomic neuroregulatory control. Blood glucose is linked to insulin secretion and can influence myocardial K^+^ conductance [15]. Perturbations in myocardial potassium ion handling alter the cardiac action potential and produce ECG morphological changes [16,17,18]. Blood glucose also affects autonomic regulation of the heart [19,20]. HRV data from ECG can be a valuable index of autonomic modulation [21]. Hyperglycemia stimulates insulin release and may directly or indirectly modify cardiac autonomic control [22,23].

Various approaches leveraging physiological signals have been proposed to estimate changes in blood glucose, using photoplethysmography (PPG) [9,11,24], electroencephalography (EEG) [10], and ECG [10,11,14,25,26,27,28]. In particular, ECG-derived features have been reported to correlate with blood glucose variability across numerous studies, motivating the development of diverse ECG-based analytic methods [17,29,30,31,32,33,34]. Representative features include fiducial-point-based features such as the QT interval, RT amplitude ratio, PR segment, ST segment, and heart rate. Reported estimation or detection techniques include density-based clustering [27], principal component analysis [25], machine-learning methods [9,11,35], particle swarm algorithm [36], and neural networks [14,27,28]. However, the extraction of ECG features associated with blood glucose is highly sensitive to signal quality and measurement resolution, making reliable acquisition essential and limiting deployment in daily-life settings.

To overcome the limitations of ECG feature extraction, artificial intelligence (AI)-based methods have increasingly leveraged ECG and HRV to estimate and detect blood glucose changes. Porumb et al. proposed a nocturnal hypoglycemia detection method using heartbeat-level ECG waveforms, which is based on a convolutional neural network (CNN) and a recurrent neural network (RNN) in a personalized, precision-medicine framework [14]. With a 10-min majority-voting window, the CNN model achieved a mean sensitivity of 87.5% and specificity of 81.7%, and the CNN + RNN model achieved a mean sensitivity of 87.5% and specificity of 84.5%. Dave et al. monitored 12 individuals with type 1 diabetes mellitus (T1DM) for 14 days and fused heartbeat-level ECG morphological features with 1-min HRV features to detect hypoglycemia and hyperglycemia at 1-min resolution [26]. Using a multi-threshold fusion model centered on the standard clinical cut-offs of 70 mg/dL (hypoglycemia) and 180 mg/dL (hyperglycemia), they reported AUCs of 0.75 and 0.78, respectively. Li et al. performed signal-level fusion of ECG and PPG by extracting time-statistical features, such as entropy, fractal measures, and mobility, as well as spatial morphological features, including ResNet-derived deep features [27]. They then applied a Choquet-integral multimodel decision fusion. The method achieved an RMSE of 1.49 mmol/L, demonstrating that the incorporation of complementary temporal and spatial information enhanced the accuracy of noninvasive blood glucose monitoring.

However, previous studies on ECG- and HRV-based glucose detection, while demonstrating the feasibility of noninvasive monitoring, exhibit several limitations. First, most investigations assumed that electrocardiographic changes occur concurrently with glucose fluctuations after compensating only for the approximately 5-min delay inherent to CGMs, without adequately accounting for autonomic nervous system responses or interindividual latency. Second, although associations between blood glucose and HRV have been noted in some reports, many studies either did not analyze this relationship or modeled blood glucose using ECG-only metrics, limiting physiological interpretability. Third, data splitting that assigns temporally adjacent ECG beats, which are drawn from short segments with near-constant glucose, to both the training and test sets yields optimistic accuracy because the beats and glucose states are nearly identical. This leakage may inflate performance through temporal autocorrelation and reduce generalizability.

Building on these considerations, we quantitatively evaluate whether ECG- and HRV-derived indices accurately reflect actual changes in blood glucose when temporal delays and participant-specific responses are taken into account. We selected participants with minimal latency between HRV and blood glucose patterns and used them for analysis, designing a classifier under conditions that minimize latency effects.

## 2. Materials and Methods

### 2.1. Experimental Protocol

The experimental protocol for this study was approved by the Institutional Review Board of Hanyang University (Approval Number HYUIRB-202306-016).

All participants were male adults aged ≥23 years (30.2 ± 11.8). Of the 30 participants, 25 were healthy subjects without physical or psychological conditions, four had type 2 diabetes mellitus (T2DM), and one was pre-diabetic. We obtained glycated hemoglobin (HbA1c) levels for all participants. Healthy subjects had HbA1c < 5.7%, pre-diabetic participant 5.7–6.4%, and participants with diabetes > 6.4% [37].

**Figure 1:**
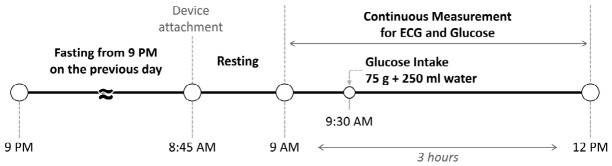
Experimental protocol of this study.

Figure 1 represents the experimental protocol. All subjects fasted from 21:00 on the day before the experiment and began measurements at approximately 09:00 in the fasting state. After a brief rest, baseline ECG and blood glucose were recorded under resting conditions. The session lasted approximately 3 h. At 30-min after initiation, participants ingested a solution containing 75 g of glucose dissolved in 250 mL of water as part of an oral glucose tolerance test (OGTT) [38]. The OGTT is a clinically standardized protocol widely used to characterize glucose tolerance under fasting conditions, providing a controlled and reproducible glucose excursion. For biomedical signal acquisition, we used a Holter ECG recorder (CardeaSolo, Cardiac Insight Inc., Bellevue, WA, USA) and a CGM device (Dexcom G6, Dexcom Inc., San Diego, CA, USA). Blood glucose was sampled every 5 min, and an ECG was recorded at 250 Hz.

Data segments with signal loss or markedly poor quality were excluded from analysis. In addition, recordings in which R-peak detection for HRV analysis could not be achieved, despite algorithmic automatic detection and manual correction, were omitted from the final dataset.

### 2.2. Signal-Processing

Data segments were screened according to the signal quality index (SQI) provided by ECG Holter, which determined their inclusion or exclusion. The ECG recordings were preprocessed using a band-pass filter (scipy 1.12.0) with a frequency range of 1–40 Hz [39,40]. To correct for an approximately 15-min delay inherent to the CGM, we temporally synchronized the CGM data with the ECG Holter time base. ECG amplitudes were converted to millivolts at the device level.

R-peak detection was performed using either Neurokit2 or the Pan–Tompkins algorithm, selecting for each recording the method that yielded superior performance, followed by manual correction [41,42]. ECG signals were segmented into 2-min windows (120-s at 250 Hz, 30,000 samples per window). For each window, R-R intervals were extracted and time-domain HRV metrics were computed (Table 1). In the same window, frequency-domain HRV metrics were obtained using the Lomb–Scargle periodogram (LSP), which mitigates distortions introduced by interpolation or resampling [43,44]. Nonlinear and statistical indices were additionally calculated (Table 1).

Because the extracted indices exhibit inter-individual differences in scale, we applied normalization to enhance comparability across individuals. Metrics such as SDNN, RMSSD, SD1, and SD2 were normalized by each subject’s mean NN (inter-beat interval), as defined in Equation (1) [45].

(1) $$Normalized\;value=\frac{Raw\;value}{Mean\;NN}\times 100$$

In contrast, mean NN, median NN, and the nonlinear indices were normalized using z-score normalization to account for subject-wise distributional differences [46], as shown in Equation (2).

(2) $$z=\frac{x-\mu }{\sigma }$$

Here, x denotes the raw value of a given index, μ the subject-specific mean of that index, and σ its standard deviation.

For deep learning-based morphology feature extraction, we performed beat segmentation within each 2-min window of the ECG. Using every R-peak as the fiducial point, each heartbeat was defined from 80 samples (320 ms) before the peak to 120 samples (480 ms) after the peak [47,48]. To maintain a consistent model input shape, the number of heartbeats per window was fixed to the minimum observed across participants. For each 2-min window, the extracted HRV indices and the segmented beat traces were paired with the temporally nearest blood glucose measurement. Blood glucose was then binarized as euglycemia or hyperglycemia using 180 mg/dL as the threshold [49].

### 2.3. Comparison Between HRV and CGM Trends

Previous studies report marked interindividual heterogeneity in the coupling between heart rate variability and glycemia [50,51,52]. In insulin-treated women with type 2 diabetes, HRV declines after meals in those with normal autonomic function, whereas patients with cardiovascular autonomic neuropathy show blunted postprandial reductions. In parallel, antecedent nocturnal glucose fluctuations are also related to subsequent daytime HRV, indicating nonsynchronous dynamics [50]. Among people with type 1 diabetes and impaired awareness of hypoglycemia, hypoglycemia-induced HRV changes are present in the majority but not in all individuals [51]. Autonomic function further shapes glycemic dynamics at the population level, as lower HRV is associated with a lower composite beta-cell response during an oral glucose tolerance test [52]. Motivated by this heterogeneity, the present study aligns HRV and CGM time series via cross-correlation to estimate person-specific lags and cluster participants by similarity in delay and coupling features. This stratification was introduced to examine ECG/HRV-based detection under conditions of reduced temporal dissociation between autonomic and glucose-related dynamics.

To compare temporal trends between HRV indices and the CGM trace, HRV indices were computed in 2-min windows, as in the model input, but with a 1-min overlap to obtain finer temporal resolution [53]. Since the CGM glucose curve changes relatively slowly, we applied locally weighted scatterplot smoothing (LOWESS) to the HRV trajectories to render smoother trends [54,55]. For each participant, the smoothed HRV time courses and the CGM glucose graph were then visualized to assess temporal covariation.

To quantify their relationship, we computed the cross-correlation between the two time series data [56], as defined in Equation (3).

(3) $$r_{xy}\left(k\right)=\frac{\sum_{t}\left(x_{t}-\bar{x})(y_{t+k}-\bar{y}\right)}{\sqrt{\sum_{t}{\left(x_{t}-\bar{x}\right)}^{2}\sum_{t}{\left(y_{t+k}-\bar{y}\right)}^{2}}}$$

Here, $x_{t}$ denotes the HRV index at time t, $y_{t+k}$ the CGM series shifted by lag k, and $\bar{x}$, $\bar{y}$ the respective series means.

From this analysis, we identified the temporal lag corresponding to the maximum correlation coefficient and used it to group subjects by response type. Importantly, this lag is estimated from each participant’s OGTT recording via cross correlation rather than assumed a priori and is used to characterize interindividual delay patterns for subgrouping analyses. In particular, we computed the cross-correlation between the CGM trace and a representative HRV index, namely the mean NN interval, and selected the correlation with the most considerable absolute value r for statistical testing. Significance was assessed at α=0.05 with N=23 by computing the t-statistic, and the resulting correlation was evaluated for statistical significance [57]. We used mean NN as the representative HRV trajectory for lag estimation because it is a fundamental time-domain descriptor of average heart period and can be computed robustly over short windows, yielding a stable series suitable for cross-correlation-based alignment.

### 2.4. Temporal Split Based Validation

To minimize temporal autocorrelation and cross-subject data overlap over time, and to obtain a realistic assessment of generalization, we used a personalized temporal-split five-fold cross-validation.

**Figure 2:**
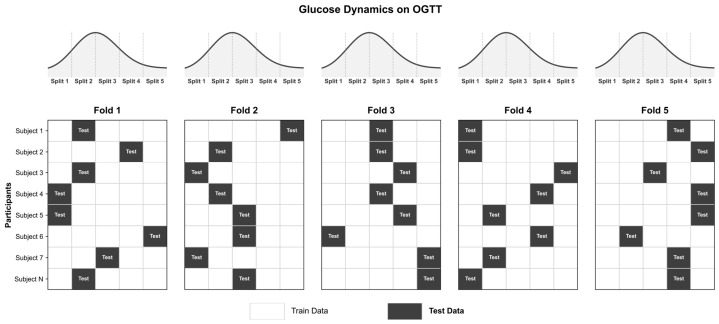
Example of the temporal-split five-fold cross-validation.

For each participant, approximately 3 h of biosignal data were acquired and temporally ordered, then partitioned into five equal-length blocks. In each fold, one block per participant was randomly assigned to the test set, and the remaining four blocks were assigned to the training set. The same procedure was applied across all subjects so that, in every fold, one-fifth of each subject’s data contributed to the test set and the remaining four-fifths to the training set. Block selection was performed without replacement across folds, resulting in five iterations where every block served as test data once. Figure 2 shows an example of the temporal split.

This design preserves within-subject temporal continuity and the class distribution. It also ensures that test-set samples are excluded from the training set, allowing for a reliable assessment of model performance in realistic scenarios.

### 2.5. Model for Blood Glucosed Level Detection

We designed a dual-input deep learning architecture that jointly leverages ECG waveform morphology and HRV indices. All preprocessing, feature extraction, and model training/evaluation were implemented in Python (v3.9.18) using TensorFlow (v2.9.1) [58], and standard scientific libraries such as bioread (v3.0.1), keras (v2.9.0), neurokit2 (v0.2.7), numpy (v1.26.4), pandas (v2.2.1), scikit-learn (v1.4.1), scipy (v1.12.0), and executed on NVIDIA GPUs to improve computational efficiency.

#### 2.5.1. Input Data Representation

The model processes two inputs in parallel. The first input comprises heartbeat-level segments extracted from each 2-min window of the ECG. To ensure consistency across subjects, the number of heartbeats per window was fixed to the minimum number observed in the dataset, and each segment spans 200 samples (approximately 0.8 s at 250 Hz). The second input contains 31 HRV indices computed from the 2-min window, the same as the beat segment input, each represented as a scalar. The two streams are processed by separate network branches and fused at the final stage.

#### 2.5.2. Network Architecture

The ECG beat segment branch was designed to learn both local morphological patterns and temporal dependencies across consecutive beats. For each segment, we applied a TimeDistributed 1D Convolution to extract local features. Convolutional layers utilized 32 filters with kernel sizes of three and five in combination, and some layers employed batch normalization, and a rectified linear unit (ReLU) activation [59,60,61]. To improve generalization, we inserted dropout (rate 0.2) and max-pooling (pool size 2) [62,63]. The resulting representations were then passed through a bidirectional long short-term memory (LSTM) architecture with 64 hidden units to capture sequence-level dynamics across the beat segments [64]. A fully connected layer with 32 units produced the ECG embedding. The HRV branch followed a simpler design: 31 indices served as input to a fully connected layer with 32 units, followed by batch normalization, yielding a compact low-dimensional representation [65].

Outputs from the two branches were concatenated and passed through a fusion module comprising fully connected layers with 64, 32, and 16 units. Batch normalization and ReLU activations were applied to the intermediate layers. Finally, a softmax layer with two units produced class probabilities for euglycemia and hyperglycemia [66].

#### 2.5.3. Training Procedure

Training used the Adam optimizer with a learning rate of 0.001 [67]. To mitigate class imbalance, we adopted focal loss [68]. The batch size was 32. We trained for up to 1000 epochs and applied early stopping when the loss failed to improve by at least 0.001 for 30 consecutive epochs [69]. Evaluation metrics included accuracy, precision, sensitivity, specificity, F1-score, and area under the receiver operating characteristic curve (AUC-ROC) [70].

### 2.6. Interpretability Techniques

We applied a gradient-based saliency map to examine which components of the ECG beat-morphology input most strongly influenced the classifier’s decisions [71]. This method defines the saliency score for each input sample as the absolute gradient of the pre-softmax logit with respect to that sample, providing a first-order approximation of feature importance via difference. A higher saliency at a given segment indicates that an infinitesimal perturbation of that segment would induce a larger change in the class logit. This enables direct visualization of how the model exploits specific beat-morphology patterns in the ECG during classification. Formally, for a class c with a logit $y_{e}$ and an input component $x_{i}$, the saliency score $S_{i}$ is $S_{i}=\left|\frac{\vartheta y_{c}}{\vartheta x_{i}}\right|$.

We also assessed the importance of the HRV-input features using feature masking analysis [72]. The approach compares the model output before and after masking a specific input component: a meaningful decrease in the output indicates that the masked component contributes substantially to computation. In this study, we removed one HRV index at a time and measured the change in the model output, thereby quantifying the relative contribution of each index to classification performance. This analysis identified which HRV indices the model relied on during learning. Formally, for model output f(x) given the original x and $f(x_{\backslash j})$ given the input with the j-th feature masked, the importance estimate $∆S_{j}$ is $∆S_{j}=f\left(x\right)-f(x_{\backslash j})$.

## 3. Results

### 3.1. Comparison of HRV Trends with CGM Glucose Curve

**Figure 3:**
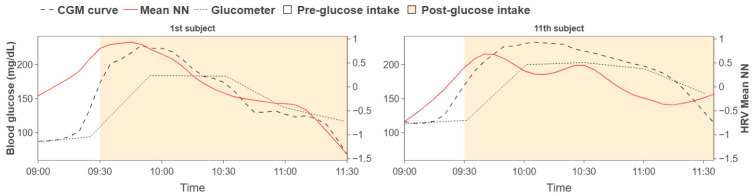
Patterns comparison between HRV trajectories and CGM curve.

Following Section 2 and Section 3, we visualized, for each participant, approximately 180 min of HRV indices alongside the CGM curve for qualitative comparison. Similar rises and falls were observed between the HRV trajectories and the CGM trace. Representative examples are shown in Figure 3, and blood glucose values measured every 30 min using a glucometer (Accu-Chek Instant®, Roche Diabetes Care, Indianapolis, IN, USA) were also presented.

To quantify temporal co-variation, we computed the cross-correlation between HRV indices and CGM while varying the temporal lag for each participant. For each participant, the lag yielding the maximum correlation coefficient was extracted and summarized in Table 2.

To assess the statistical significance of the maximum correlation coefficient, we applied the *t*-test for Pearson’s r. For the relationship between the mean NN interval and the CGM trace, with N=23 and r=0.651, the t-statistic was t=3.93, which exceeds the two-tailed critical value at α=0.05 ($t_{\left(21\right)}=2.08$). These results indicate a statistically significant association between HRV and CGM glucose.

**Figure 4:**
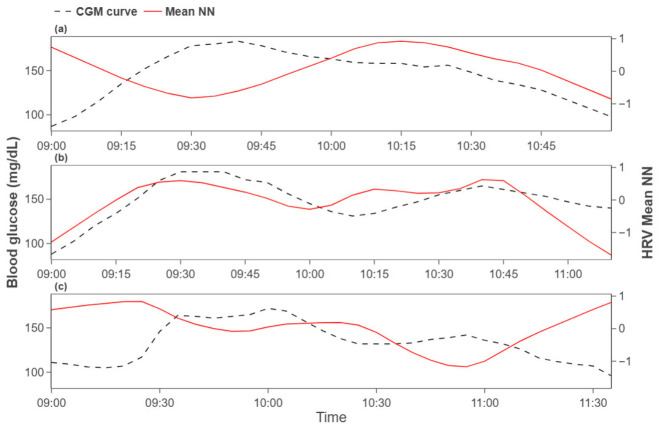
Group-wise patterns by temporal lag category (**a**) lag<−4 group, (**b**) −4≤lag≤4 group, and (**c**) lag>4 group.

Participants were then grouped by the temporal lag at which the maximum correlation occurred: (i) lag<−4, (ii) −4≤lag≤4, (iii) lag>4. We focused subsequent analyses on group (ii), which exhibits minimal latency between HRV and glucose responses. Figure 4 illustrates representative groupwise trends. This grouping highlights heterogeneous delay patterns across participants, with the short-delay group suggesting a more sensitive reflection of glucose dynamics in HRV indices.

### 3.2. Performance of the Hyperglycemia Detection Model

We performed temporal-split five-fold cross-validation by partitioning each participant’s 3-h recording into five sequential blocks and, in turn, assigning each block as the test set. The average classification performance across folds was as follows: accuracy 0.892±0.071, precision 0.763±0.189, sensitivity 0.858±0.189, specificity 0.915±0.055, F1-score 0.791±0.159, AUC-ROC 0.887±0.101.

Across the temporal-split five-fold evaluation, the normal blood glucose class consistently showed a high true-negative rate. The true-positive rate for the high blood glucose class varies by fold: detection was relatively lower in folds one and two, whereas folds four and five exhibited distinctly higher detection rates.

Table 3 summarizes fold-wise classification accuracies. First fold was 0.855, second fold was 0.787, third fold was 0.918, fourth fold was 0.956, and fifth fold was 0.946. The overall mean accuracy was 0.892. Performance varied across folds but remained stable overall.

### 3.3. Ablation Study

We conducted an ablation study to quantify the contribution of each input component. Three configurations were compared: dual input using both ECG beat segments and HRV indices, ECG-only input, and HRV-only input. Detailed performance metrics are reported in Table 4.

The dual-input model achieved the best results with an accuracy of 0.893, F1-score of 0.789, and AUC-ROC of 0.867. Using ECG beat segments alone reduced performance to an accuracy of 0.716, F1-score of 0.607, and AUC-ROC of 0.777, indicating that morphology alone is insufficient for stable classification of blood glucose states. Using HRV indices alone also resulted in degraded performance, with an accuracy of 0.785, an F1-score of 0.595, and an AUC-ROC of 0.800. These findings demonstrate that ECG- and HRV-derived information is individually limited but complementary, and that the dual-input design enhances both accuracy and generalizability.

### 3.4. Analysis of Model Attention and Feature Contributions

#### 3.4.1. Gradient-Based Saliency Map for ECG Beat Morphology

**Figure 5:**
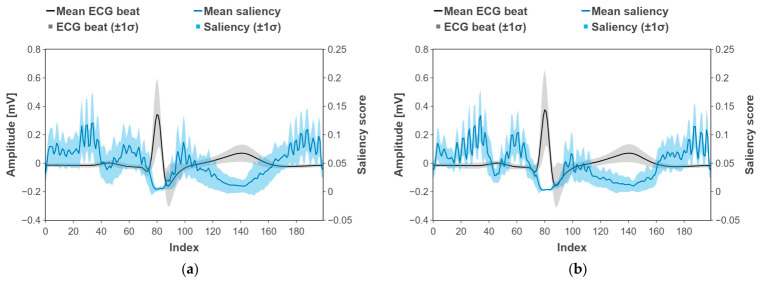

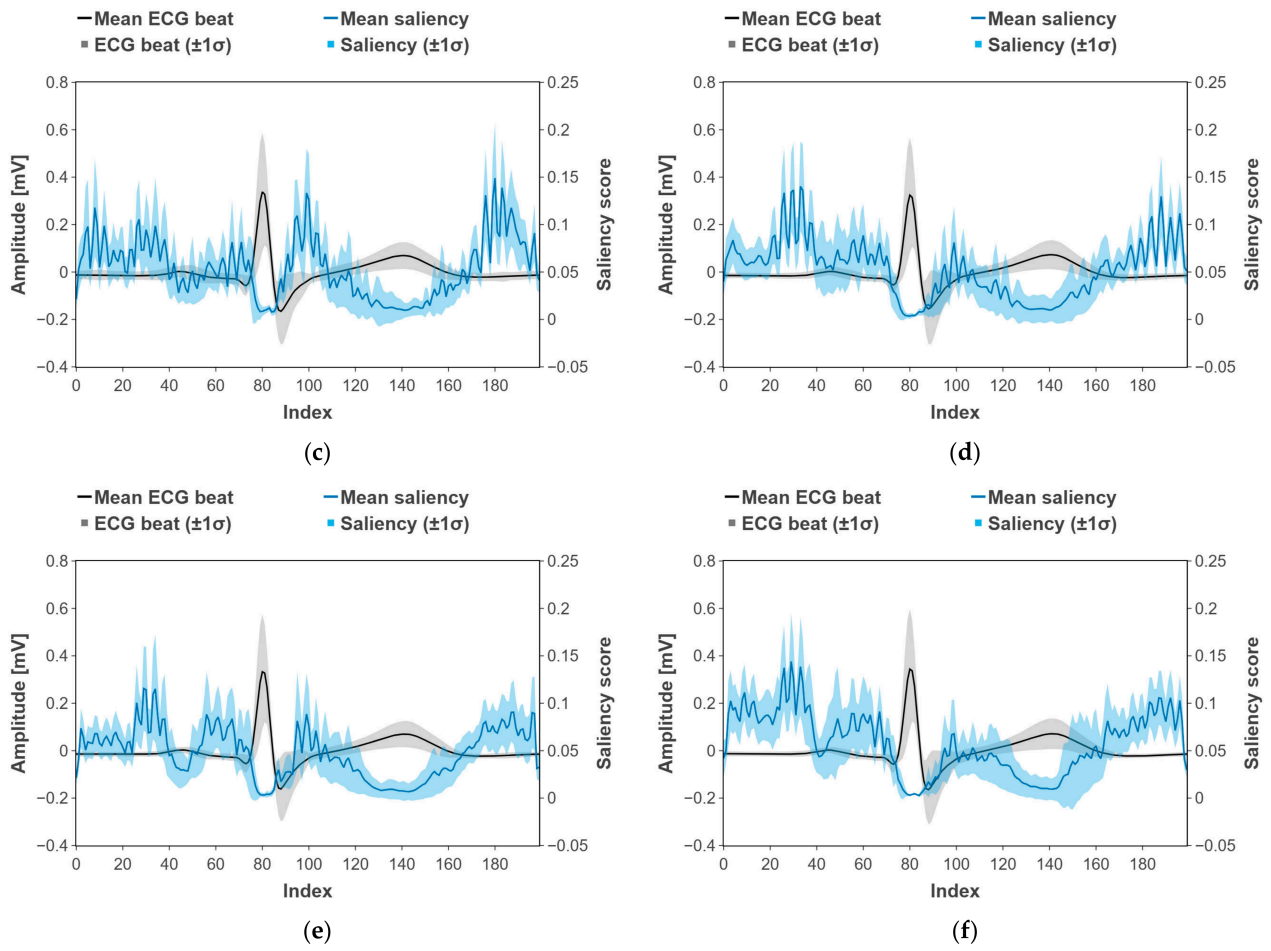
Test-set-averaged gradient-based saliency map for ECG beat morphology. (**a**) Total folds, (**b**) first fold, (**c**) second fold, (**d**) third fold, (**e**) fourth fold, and (**f**) fifth fold.

Figure 5 illustrates the average ECG beat waveform, along with its gradient-based saliency profile, for each fold and total fold. The black trace denotes each participant’s mean ECG beat, and the light-black band indicates ±1 standard deviation. The blue trace denotes the mean saliency score, and the light-blue band indicates ±1 standard deviation.

In both cases, the saliency for logit of the hyperglycemia as a positive class was highest around the onset and offset of the P wave and immediately after the T wave. Elevated saliency also appeared from just after the QRS complex to the onset of the T wave, and in the post-T segment. In contrast, saliency was lower within the QRS complex itself and within the T wave. These patterns were consistent across participants and suggest that the model relies on the boundaries of fiducial waves in the ECG, particularly transitions near the P and T waves, to inform its decisions.

#### 3.4.2. Feature Importance Analysis of HRV Indices

**Figure 6:**
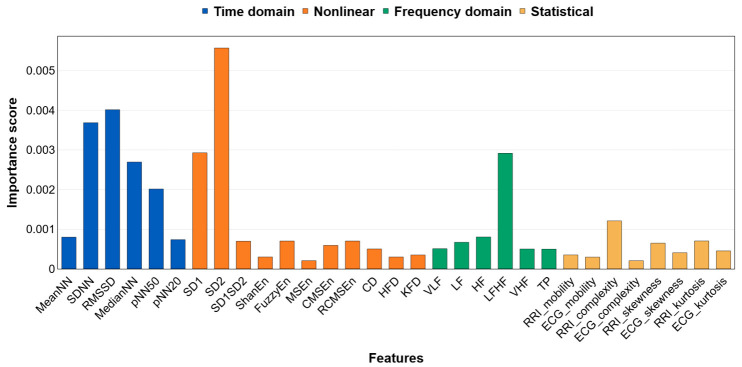
Feature masking importance for HRV indices.

Figure 6 presents the mean HRV feature importance obtained across five-fold cross-validation. SD2 showed the highest contribution at approximately 0.00557, followed by RMSSD at approximately 0.00402, SDNN at approximately 0.00369, SD1 at approximately 0.00292, median NN at approximately 0.00271, and pNN50 at approximately 0.00200. In the frequency domain, the LF/HF ratio reached approximately 0.00291 and exceeded the other spectral metrics, VLF, LF, and HF. Entropy-based measures, including Shannon entropy, FuzzyEn, MSEn, CMSEn, and RCMSEn, were all at or below 0.001. Fractal metrics HFD and KFD, as well as statistical descriptors such as ECG skewness and kurtosis, were all below 0.0015, indicating a limited contribution.

## 4. Discussion

Table 3 reports fold-specific performance of the hyperglycemia detection model. The average sensitivity was 0.858±0.189 and the average specificity was 0.915±0.055, exceeding previously reported ECG-based results [14,26]. Performance varied across folds. First fold achieved sensitivity 0.923 and specificity 0.843, consistent with prior ECG-based reports [14]. In contrast, the second fold showed noticeably lower accuracy and sensitivity. This likely reflects temporal splits: in the second fold, a relatively large number of participants had their third temporal block, the interval encompassing the densest hyperglycemic phase of the OGTT procedure, assigned to the test set. Consequently, the training set contained fewer hyperglycemic samples, limiting the model’s ability to learn hyperglycemia-related patterns [Table 5]. The reduced sensitivity in fold two likely reflects fold-specific class composition under the temporal split. We therefore provide fold-wise and subject-wise window counts together with subject-wise test accuracies in the Supplementary Materials (Tables S1 and S2) to quantitatively document this effect. This suggests that temporal splitting, although realistic, can yield fold-to-fold variability depending on the data volume and split configuration.

Despite this, folds 3–5 exhibited consistently stable performance, and each fold outperformed prior reports [14,26]. These results suggest that the proposed model maintains reliable performance under limited data and conservative temporal splits. Given that prior studies often evaluated repeated short segments that can inflate performance, the present approach demonstrates meaningful accuracy under more realistic validation conditions.

In addition, the results in Section 3.3 showed that ECG morphology and HRV indices act in a complementary manner. Accuracy dropped to 0.716 with ECG-only input and to 0.785 with HRV-only input, whereas the dual-input model reached 0.893. This indicates that myocardial conduction features and autonomic responses are both key cues for detecting hyperglycemia and that an integrated model is more clinically plausible than single-signal approaches.

**Figure 7:**
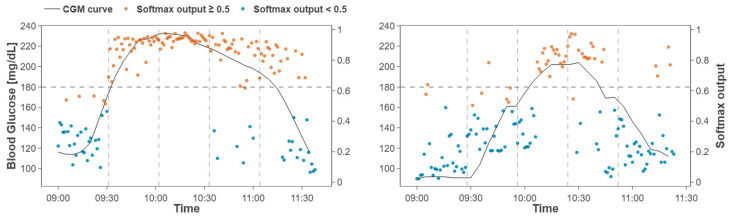
Temporal alignment between CGM curve and an out-of sample probability trace for two specific subjects obtained by concatenating fold-wise test segments during OGTT.

Consistency with the OGTT curve was also observed. For two representative participants, the softmax output of the model based on ECG and HRV exhibited temporal fluctuations similar to the CGM glucose trace (Figure 7). In the first participant, the model output rose nearly synchronously around the time the glucose level crossed 180 mg/dL and remained high throughout the hyperglycemic interval. These observations suggest that ECG and HRV indices can reflect glucose dynamics and that the proposed model effectively captures acute glucose excursions.

Figure 5 illustrates the salient regions of ECG segment samples identified by the deep learning model during hyperglycemia detection. The gradient-based saliency map for the logit of the hyperglycemia as a positive class showed the highest values around the onset of the P wave and immediately after the T wave. Elevated saliency was also observed between the end of the QRS complex and the onset of the T wave, as well as in the post-T segment. In contrast, saliency was lower within the T wave itself and within the QRS complex.

Well-established markers of heterogeneity in ventricular repolarization include the corrected QT interval (QTc) and QT dispersion [73]. Prior studies have shown that acute hyperglycemia or hyperinsulinemia increases QTc and QT dispersion. In patients with T2DM, prolongation of the T-peak to T-end interval and QT interval has been reported, indicating exacerbated repolarization heterogeneity [74,75]. Marfella et al. demonstrated that induction of acute hyperglycemia in healthy subjects significantly increased QTc, QTc dispersion, and the PR interval, findings that align with the present saliency map results highlighting the boundaries of the P wave and PR interval [74]. Similarly, Taubel et al. reported that hyperglycemia and chronic metabolic abnormalities in patients with diabetes were associated with QTc prolongation, which may contribute to repolarization abnormalities and an elevated risk of ventricular arrhythmia [75]. These findings support the observation that the post-T wave segment is an important discriminative region.

Indices related to atrioventricular conduction include P wave duration and the PR interval. Prior studies have reported significant prolongation of these indices in T2DM [76,77]. Demir et al. found that maximum P wave duration and P wave dispersion were significantly increased in patients with T2DM compared to controls, correlating with left atrial enlargement (LAE) and atrial electromechanical delay [76]. Consistently, Akyel et al. observed significant PR interval prolongation in diabetic patients, which was interpreted as evidence of atrial conduction delay [77]. These findings suggest that disturbances in atrioventricular conduction arise in the context of impaired glucose metabolism, in agreement with the saliency map results, which emphasize the boundaries of the P wave and PR interval.

According to feature masking analysis (Figure 6), SD2 showed the highest importance, followed by RMSSD, SDNN, SD1, median NN, and pNN50; in the frequency domain, the LF/HF ratio exhibited a comparatively strong contribution. These patterns suggest that the model increases the hyperglycemia logit by leveraging long-term variability, short-term variability, and sympathovagal balance indices. Since the importance scores were computed by removing one HRV index at a time and measuring the resulting drop in output, they more directly reflect the causal contribution of each index to the model’s decision.

RMSSD and SD1 are established markers of short-term variability and vagal (parasympathetic) activity, whereas SDNN and SD2 reflect longer-term, overall variability [53,78,79]. In this context, the jointly high importance of RMSSD and SD1, together with SDNN and SD2 supports the view that both short- and long-timescale ANS fluctuations contribute to hyperglycemia classification. The prominent contributions of pNN50, median NN, and mean NN indicate that distributional properties of inter-beat variability and the tonic level of heart rate were also exploited [78]. Consistent with RMSSD, pNN50 indexed short-term variability and parasympathetic tone [78].

The LF/HF ratio is commonly interpreted as an index of sympathovagal balance [78,80]. A relatively high importance score does not, in itself, demonstrate that sympathovagal balance is the decisive cue for classification, and this interpretation has known limitations [81]. Even so, the result suggests that the relative spectral power in the low- and high-frequency bands contributed, in combination with other HRV indices, to hyperglycemia prediction.

Unlike the indices above, entropy- and fractal-based measures exhibited very low feature-masking importance. This likely reflects the limited interpretability and reduced reliability of nonlinear metrics when computed within a 2-min window [53].

The importance profile in Section 3.4.2 is consistent with prior evidence that OGTT-induced acute hyperglycemia perturbs the ANS. Reports indicate that OGTT elevates glucose and alters ANS and microvascular reactivity [82]. Baseline HRV has been shown to predict OGTT outcomes [83]. Impaired ANS control has also been discussed even at high-normal glucose levels [23]. Taken together, these findings and the present results suggest that ANS modulation during hyperglycemia is captured in HRV and used by the model as a cue for classification.

This study has several limitations. First, because we analyzed only 3 h of short-term monitoring, the data are insufficient to evaluate long-term or delayed effects of hyperglycemia on HRV and ECG. Future work should employ 24-h or multi-day continuous monitoring to characterize subject-specific latency and autonomic responses with greater precision. Second, prior studies indicate that cardiovascular responses to glucose metabolism may vary in relation to circadian rhythm. Our data were collected exclusively in the morning, which limits interpretation across different times of day [84]. Both glucose dynamics and autonomic cardiovascular regulation exhibit circadian structure, and wearable time-series studies model circadian components in glucose and their relationships with ANS-related signals, HRV–glucose coupling may vary by time of day, this should be tested in future mixed-sex, repeated/ambulatory studies [84,85,86,87,88]. Because classification analyses focused on participants with minimal HRV–glucose latency to reduce temporal misalignment, performance and generalizability to individuals with larger or context dependent delays require further validation. Thus, the reported performance should be interpreted within this physiologically constrained subgroup, and future work should develop designs and models that accommodate heterogeneous, context-dependent lags without subgroup restriction. Third, the cohort size was modest and restricted to male adults, which may limit external validity across sex, age, and broader clinical phenotypes. Although prior reports have found no significant sex differences in acute glucose and insulin responses, broader external validity will require follow-up studies encompassing diverse sex, age, and clinical profiles [85,86,87,88]. Fourth, this study is that the experimental design relied exclusively on OGTT-induced glucose excursions without a matched control condition, such as ingestion of an isovolumetric non-glucose solution or an isocaloric alternative. Accordingly, our model should be interpreted as detecting an OGTT-associated hyperglycemic state under controlled conditions rather than demonstrating glucose-specific physiological sensing. Oral glucose ingestion triggers multiple physiological processes beyond the rise in glucose itself, including gastrointestinal activation, insulin secretion, satiety-related responses, possible drowsiness, and time-dependent autonomic modulation during prolonged seated rest [89,90,91,92,93]. Accordingly, the model should be interpreted as detecting an acute OGTT-associated hyperglycemic state under controlled conditions rather than demonstrating specificity to elevated glucose alone. Lastly, because prior work and our cross-correlation analysis indicated marked interindividual heterogeneity in HRV–glucose temporal coupling, the modeling analysis focused on a physiologically stratified subgroup with relatively small lags [50,51,52]. This design allowed us to examine whether ECG and HRV contain detectable information associated with the hyperglycemic phase under conditions of reduced temporal dissociation. However, the resulting performance should be interpreted within this restricted subgroup rather than as population-level performance, and future studies will need to develop approaches that accommodate such heterogeneity without subgroup restriction. Taken together, these limitations suggest that the present findings are best interpreted within the context of a controlled OGTT setting. While the results support the feasibility of ECG- and HRV-based detection of the hyperglycemic phase under these conditions, further studies will be needed to clarify the extent to which the detected signatures are specific to glucose elevation itself and to determine how well the approach generalizes to broader populations and real-world settings.

In summary, the dual-input model outperformed single-signal approaches, demonstrating that myocardial conduction features and autonomic variability act in a complementary manner in hyperglycemia detection. The saliency analysis highlighted boundaries of ECG waves, and the feature-masking analysis identified SD2, RMSSD, and the LF/HF ratio as key HRV cues. These findings align with physiological evidence that acute hyperglycemia elicits both repolarization abnormalities and ANS changes. The subject grouping, based on individual responses, further improved analytical resolution and predictive performance. Taken together, the results indicate that noninvasive biomedical signal-based hyperglycemia detection is not merely predictive but physiologically grounded, with potential for translation to wearable platforms for early diabetes management and risk stratification.

## 5. Conclusions

This study demonstrates the feasibility of detecting hyperglycemia noninvasively by analyzing HRV and ECG signals. We addressed key challenges in prior ECG-based glucose studies by accounting for individual HRV–glucose delays and combining autonomic and cardiac features in a dual-input deep learning model. Under a realistic, temporally segmented evaluation, our model achieved ~89% accuracy in distinguishing hyperglycemic episodes, substantially outperforming single-input models that used only ECG or only HRV. These results suggest that myocardial conduction features and autonomic variability interact in a complementary manner during acute glucose elevations. Gradient-based saliency mapping further showed that the classifier focused on specific ECG waveform regions, especially around the P-wave and T-wave boundaries. At the same time, feature masking identified classical HRV metrics (SD2, RMSSD, LF/HF) as the dominant contributors. Notably, the model’s attention aligned with known physiological markers, suggesting that glucose-induced perturbations in cardiac repolarization and autonomic tone were being captured.

Our findings carry implications for both physiological understanding and practical glucose monitoring. The observed HRV response to hyperglycemia underscores those autonomic fluctuations play a significant role in glucose regulation and should not be overlooked in future models of glycemic prediction. In line with evidence that OGTT perturbs the autonomic nervous system, our results confirm that acute hyperglycemia evokes ANS modulation, as measured by HRV, which can enhance detection when combined with ECG morphology. However, because the study did not include a matched non-glucose control condition, the observed ECG/HRV signatures cannot be attributed uniquely to elevated glucose itself and may reflect a composite physiological response to glucose ingestion. Future studies incorporating appropriate control interventions will be necessary to determine the extent to which the detected signatures are specific to hyperglycemia per se. In addition, the present findings should also be interpreted in light of the lag-based subgroup selection used in this study. This study reflects feasibility in a physiologically selected subgroup rather than established performance in an unselected population. Future work should test whether similar performance can be achieved without excluding subjects and whether individualized lag-aware modeling can improve generalizability. Despite this limitation, by leveraging ubiquitous wearable ECG sensors, this strategy could enable early identification of dysglycemia in at-risk populations without invasive measurements. Future studies should validate these findings in larger, more diverse, and unselected cohorts, incorporate matched-control interventions to better assess glucose-specific effects, and extend monitoring to 24-h or multi-day periods and to real-world settings where glucose fluctuations are not tied to controlled ingestion. In addition, integrating the dual-input model into real-time wearable platforms will be an important step toward translating it into daily diabetes prevention and management.

## Figures and Tables

**Table 1:** HRV indices and statistical features used in this study.

Domain	Parameter	Definition
Time domain	MeanNN	Mean of normal-to-normal (NN) intervals
	SDNN	Standard deviation of NN intervals
	RMSSD	Root mean squared differences between adjacent RR intervals
	pNN50	Percentage of successive RR intervals differing by more than 50 ms
	pNN20	Percentage of successive RR intervals differing by more than 20 ms
	MedianNN	Median of normal-to-normal (NN) intervals
Nonlinear domain	SD1	Short-term variability based on Poincaré plot
	SD2	Long-term variability based on Poincaré plot
	SD1/SD2	Ratio of short-term to long-term variability
	ShanEn	Shannon entropy of RR intervals (signal uncertainty)
	FuzzyEn	Fuzzy entropy of RR intervals (noise-robust complexity)
	MSEn	Multiscale entropy across multiple temporal scales
	CMSEn	Composite multiscale entropy with improved stability
	RCMSEn	Refined composite multiscale entropy with better robustness
	CD	Correlation dimension of RR interval dynamics
	HFD	Higuchi fractal dimension of RR intervals
	KFD	Katz fractal dimension of RR intervals
LSP-based Frequency domain	VLF	Very low frequency power calculated by Lomb–Scargle periodogram (<0.04 Hz)
	LF	Low frequency power calculated by Lomb–Scargle periodogram (0.04–0.15 Hz)
	HF	High frequency power calculated by Lomb–Scargle periodogram (0.15–0.4 Hz)
	LF/HF ratio	Ratio of LF to HF spectral power
	VHF	Very high frequency power calculated by Lomb–Scargle periodogram (>0.4 Hz)
	Total Power	Sum of spectral power over all frequency band
Statistical Features	RRI mobility	Hjorth mobility of RR intervals
	ECG mobility	Hjorth mobility of ECG signals
	RRI complexity	Hjorth complexity of RR intervals
	ECG complexity	Hjorth complexity of ECG signals
	RRI skewness	Statistical skewness of RR interval distribution
	ECG skewness	Statistical skewness of ECG amplitude distribution
	RRI kurtosis	Kurtosis of RR interval distribution
	ECG kurtosis	Kurtosis of ECG amplitude distribution

**Table 2:** Subject-wise temporal lag at maximum HRV–CGM correlation coefficients.

Subject Number	Temporal Lag [5-min]	Correlation Coefficient	Subject Number	Temporal Lag [5-min]	Correlation Coefficient
1	0	0.88	13	−1	0.47
2	0	0.62	14	−4	0.71
3	3	0.75	15	−13	0.37
4	−15	0.54	16	−16	0.48
5	0	0.83	17	−1	0.71
6	7	0.73	18	8	0.42
7	−18	0.49	19	0	0.66
8	−14	0.62	20	4	0.67
9	5	0.67	21	0	0.66
10	−12	0.57	22	0	0.97
11	2	0.81	23	−6	0.70
12	−13	0.65	Mean	−3.65	0.65

**Table 3:** Fold-wise classification performance under temporal-split five-fold cross-validation.

	Accuracy	Precision	Sensitivity	Specificity	F1-Score	AUC-ROC
First fold	0.855	0.495	0.923	0.843	0.644	0.883
Second fold	0.787	0.691	0.528	0.898	0.599	0.713
Third fold	0.918	0.745	0.988	0.897	0.850	0.943
Fourth fold	0.956	0.906	0.966	0.950	0.935	0.958
Fifth fold	0.946	0.976	0.886	0.986	0.929	0.936
AVG	0.892	0.763	0.858	0.915	0.791	0.887
STD	0.071	0.189	0.189	0.055	0.159	0.101

**Table 4:** Ablation study comparing input configurations.

	Accuracy	F1-Score	AUC-ROC
Beat morphology only	0.716	0.607	0.777
HRV only	0.785	0.595	0.800
Morphology + HRV	0.893	0.789	0.867

**Table 5:** Temporal split composition for the second fold.

	First Split	Second Split	Third Split	Fourth Split	Fifth Split
Fold 2	3	2	5	1	1

## Data Availability

The datasets in this study are not publicly available because they contain confidential human participant data and public sharing was not included in the informed consent. To protect participant privacy and comply with ethical and legal requirements, access to the data is restricted. The data may be provided on request from the corresponding author upon reasonable request.

## Supplementary Materials

The following supporting information can be downloaded at: https://www.mdpi.com/article/10.3390/bios16050251/s1. Table S1: Subject-wise classification accuracy across cross-validation folds. Table S2: Subject-wise counts of euglycemia and hyperglycemia windows across cross-validation folds.
