# Supplementary material for: Noninvasive Detection of Acute Hyperglycemia Using Signal from Wearable ECG Sensors Considering Individual HRV Response Delays to Glucose
Source: Biosensors (Basel). 2026 Apr 29;16(5):251. doi: 10.3390/bios16050251 (PMC13205030; doi:10.3390/bios16050251)
Supplement: Supplementary file 1 [file biosensors-16-00251-s001.zip › biosensors-4231564-supplementary.pdf]

Table S1: **Subject-wise classification accuracy across cross-validation folds.** Per subject accuracy is reported for each test fold, where each value corresponds to the classification accuracy on the subject's samples included in the fold-specific test set.

Acc	Sub1	Sub2	Sub3	Sub4	Sub5	Sub6	Sub7	Sub8	Sub9	Sub10	Sub11	Sub12
Fold1	1.00	0.82	1.00	1.00	0.88	1.00	0.74	1.00	0.34	1.00	0.90	0.68
Fold2	0.63	1.00	1.00	0.94	0.79	0.30	0.84	1.00	0.65	1.00	1.00	0.41
Fold3	1.00	0.75	1.00	1.00	0.93	0.88	0.87	1.00	0.90	1.00	0.73	0.96
Fold4	1.00	1.00	0.96	1.00	0.93	0.82	1.00	1.00	1.00	1.00	0.85	0.93
Fold5	1.00	0.96	0.92	1.00	0.82	1.00	0.81	1.00	0.97	1.00	0.93	0.93

Table S2: **Subject-wise counts of euglycemia and hyperglycemia windows across cross-validation folds.** For each subject and each test fold, this table reports the number of labeled analysis windows assigned to hyperglycemia and euglycemia in the fold-specific test set.

Subject number	1	2	3	4	5	6	7	8	9	10	11	12
Hyperglycemia (fold1)	0	11	0	0	0	0	11	0	0	0	30	0
Euglycemia (fold1)	24	17	26	34	32	37	24	28	35	28	0	28
Hyperglycemia (fold2)	0	28	0	0	0	24	0	0	22	0	0	32
Euglycemia (fold2)	24	0	25	34	28	9	31	28	9	28	30	0
Hyperglycemia (fold3)	0	0	0	0	0	0	27	0	28	0	0	28
Euglycemia (fold3)	24	28	25	34	28	33	4	31	3	32	30	0
Hyperglycemia (fold4)	0	32	0	0	19	11	31	0	0	0	0	26
Euglycemia (fold4)	28	0	25	34	9	22	0	28	31	28	34	2
Hyperglycemia (fold5)	0	28	7	0	21	0	31	0	0	0	25	28
Euglycemia (fold4)	24	0	18	38	7	33	0	28	31	28	5	0
